# Supplementary material for: Transcriptome-Wide Characterization of piRNAs during the Developmental Process of European Honey-Bee Larval Guts
Source: Genes (Basel). 2022 Oct 17;13(10):1879. doi: 10.3390/genes13101879 (PMC9602049; doi:10.3390/genes13101879)
Supplement: Supplementary file 1 [file genes-13-01879-s001.zip › Table S2.pdf]

Table S2. Detailed information about the identified DEpiRNAs in *A. mellifera* larval guts.

Gene ID	log2_FC	Pvalue	Classify
piR-ame-1009988	14.52139821	7.22E-05	
piR-ame-1028819	1.034668215	0.024017822	
piR-ame-1075475	-1.19554946	0.006042626	
piR-ame-1077365	-1.649490421	0.00074854	
piR-ame-1146559	-1.039234323	0.00384788	
piR-ame-1146560	-1.346401603	0.006373339	
piR-ame-1150261	2.43097329	0.000539812	
piR-ame-1176116	-1.293388999	0.019680137	
piR-ame-1183555	2.738821322	0.005660361	
piR-ame-1184482	1.136360028	0.039990625	
piR-ame-1186994	-1.658142914	0.040683334	
piR-ame-1192181	-1.284486198	0.026096215	
piR-ame-1219967	-1.240260699	0.01507866	
piR-ame-1223398	-11.37535082	3.19E-09	
piR-ame-1233036	1.778667466	0.014847528	
piR-ame-1241456	-1.31278976	0.011073679	
piR-ame-1242256	1.079919925	0.023886915	
piR-ame-14055	14.52139821	7.22E-05	
piR-ame-14476	1.704554003	0.03793934	
piR-ame-145057	10.84556062	5.36E-08	
piR-ame-15258	1.81125685	0.032806637	Am4 vs. Am5
piR-ame-180189	2.437074481	0.000523596	
piR-ame-18387	3.704133929	0.030395754	
piR-ame-208022	3.704133929	0.030395754	
piR-ame-225379	-1.094921951	0.000269889	
piR-ame-237656	14.46023574	6.41E-05	
piR-ame-237658	3.685559249	0.02994433	
piR-ame-241618	1.573505518	0.015856361	
piR-ame-247619	2.009937572	0.00170725	
piR-ame-251803	12.09607601	0.000118641	
piR-ame-259611	1.023838421	0.023776365	
piR-ame-269855	3.731555176	0.031064073	
piR-ame-31793	14.4850127	6.73E-05	
piR-ame-320912	14.4850127	6.73E-05	
piR-ame-325111	1.250998634	0.015785532	
piR-ame-378755	2.866528181	0.003350951	
piR-ame-387266	-1.228917519	0.009897205	
piR-ame-387267	-1.069617382	0.004554813	
piR-ame-39500	1.81125685	0.032806637	
piR-ame-39501	1.81125685	0.032806637	
piR-ame-414850	3.731555176	0.031064073	

piR-ame-456655	14.52139821	7.22E-05	
piR-ame-471152	1.23395953	0.043693073	
piR-ame-47855	3.685559249	0.02994433	
piR-ame-5	14.52139821	7.22E-05	
piR-ame-500944	2.847408657	0.002291196	
piR-ame-502767	11.64686918	3.39E-05	
piR-ame-504668	2.769193153	0.013820964	
piR-ame-506264	11.45955116	1.54E-05	
piR-ame-521089	2.47564098	0.000602643	
piR-ame-605224	2.443142079	0.000529945	
piR-ame-612850	2.441380028	0.000530794	
piR-ame-633772	-1.03605132	0.003829105	
piR-ame-638412	2.034105921	0.012767334	
piR-ame-641984	2.425762878	0.00056452	
piR-ame-665593	1.601829912	0.037890966	
piR-ame-670896	3.695411797	0.007655153	
piR-ame-670897	3.075365087	0.017428252	
piR-ame-70388	2.895840386	0.003734866	
piR-ame-718308	3.35935963	0.002756793	
piR-ame-742536	11.33688401	5.42E-07	
piR-ame-748816	1.110466873	0.035827816	
piR-ame-750627	2.199810264	0.002553317	
piR-ame-784272	1.488182863	0.005844392	
piR-ame-850115	2.722232777	0.015722835	
piR-ame-852767	11.64686918	3.39E-05	
piR-ame-856650	-1.493954935	0.000289637	
piR-ame-904144	2.721687573	0.007156561	
piR-ame-918866	1.749304739	0.04146075	
piR-ame-928134	3.498401823	0.001100457	
piR-ame-930873	-1.009393368	0.042679462	
piR-ame-934193	2.009937572	0.00170725	
piR-ame-941843	2.189862052	0.009777397	
piR-ame-945352	11.43946558	4.77E-06	
piR-ame-974219	2.432755806	0.000538972	
piR-ame-978292	3.704133929	0.030395754	
piR-ame-990954	11.81263727	5.82E-05	
piR-ame-1173337	-10.963170640576	1.34E-05	
piR-ame-1243913	3.009718729	0.018774132	
piR-ame-1246710	-1.184855605	0.044872939	
piR-ame-260979	-11.19715525	3.78E-05	Am5 vs. Am6
piR-ame-31653	-10.96317064	1.34E-05	
piR-ame-358367	-11.19715525	3.78E-05	
piR-ame-592661	1.13853464	0.005496965	
